# Mesenchymal stem cell therapy alleviates the neuroinflammation associated with acquired brain injury

**DOI:** 10.1111/cns.13378

**Published:** 2020-05-01

**Authors:** Brooke Bonsack, Sydney Corey, Alex Shear, Matt Heyck, Blaise Cozene, Nadia Sadanandan, Henry Zhang, Bella Gonzales‐Portillo, Michael Sheyner, Cesar V. Borlongan

**Affiliations:** ^1^ Department of Neurosurgery and Brain Repair University of South Florida Tampa FL USA

**Keywords:** bone marrow–derived mesenchymal stem cells, clinical trials, inflammation, ischemic stroke, preclinical studies, traumatic brain injury

## Abstract

Ischemic stroke and traumatic brain injury (TBI) comprise two particularly prevalent and costly examples of acquired brain injury (ABI). Following stroke or TBI, primary cell death and secondary cell death closely model disease progression and worsen outcomes. Mounting evidence indicates that long‐term neuroinflammation extensively exacerbates the secondary deterioration of brain structure and function. Due to their immunomodulatory and regenerative properties, mesenchymal stem cell transplants have emerged as a promising approach to treating this facet of stroke and TBI pathology. In this review, we summarize the classification of cell death in ABI and discuss the prominent role of inflammation. We then consider the efficacy of bone marrow–derived mesenchymal stem/stromal cell (BM‐MSC) transplantation as a therapy for these injuries. Finally, we examine recent laboratory and clinical studies utilizing transplanted BM‐MSCs as antiinflammatory and neurorestorative treatments for stroke and TBI. Clinical trials of BM‐MSC transplants for stroke and TBI support their promising protective and regenerative properties. Future research is needed to allow for better comparison among trials and to elaborate on the emerging area of cell‐based combination treatments.

## 1 INTRODUCTION

Acquired brain injury (ABI) entails any injury that disrupts neuronal activity and is not degenerative, hereditary, congenital, or induced by birth trauma. Traditional examples of ABI include not only stroke and traumatic brain injury (TBI), but also near drowning, aneurysm, tumor, meningitis and other infections involving the brain, and injuries resulting from lack of oxygen supply to the brain, such as those seen in myocardial infarction. ABI may involve a structural insult, changes to metabolic activity, or disruption to neuronal capabilities. While progressive loss of brain cells and debilitating motor and cognitive deficits play a role in all these disorders, stroke and TBI overlap particularly closely in pathology and impose an immense burden on the American and global populations.

The American Stroke Association reports that stroke is the fifth leading cause of death in the United States, taking as many as 142 000 lives every year, and is also the leading cause of preventable long‐term disability.^1^ Moreover, the United States spends over $45 billion dollars every year on medications and healthcare services to treat and care for those affected.^1^ Stroke patients also display an increased risk of developing dementia, which, in turn, may amplify their health and economic burdens.^2^ Along with cognitive impairments, stroke patients often suffer paralysis and other physical impairments which entail exhaustive rehabilitation, contributing to stroke's high morbidity statistics.^2^, ^3^

Similarly, while less pervasive than stroke in terms of mortality, TBI caused approximately 2.4 million emergency room visits, hospitalizations, or deaths in the United States in 2010 alone.^4^ Moreover, estimates indicate that 5.3 million Americans are presently living with disabilities resulting from TBI.^4^ More recent assessments implicate TBI in approximately 82 000 deaths and 2.1 million hospital discharges yearly in Europe, and TBI is responsible for 37% of injury‐related deaths in 24 European Union countries.^5^ Hallmarks of TBI include bruising, bleeding, torn tissues, and other forms of physical damage to the brain that can lead to long‐term impairment or death. Additionally, cognitive symptoms of TBI often involve problems with memory, attention, concentration, or thinking, as well as mood or behavioral changes, fatigue or lethargy, and alterations in sleep pattern.^4^ Moreover, prior TBI is linked to increased incidence of other neurological disorders, such as Alzheimer's disease and Parkinson's disease, further increasing the long‐term costs and health ramifications.^6^, ^7^

## 2 CELL DEATH CLASSIFICATION IN ABI

As noted above, stroke and TBI share some overlapping pathologies, but are distinct from each other because stroke primarily ensues as a nontraumatic ischemic insult, whereas TBI obviously arises from a traumatic episode. Beyond these nontraumatic or traumatic events, these two ABI disorders display similar cell death features. Primary cell death may manifest as either focal or diffuse, with the former characterized by the demise of cells within a localized brain area (referred to as infarcted core and ischemic penumbra or peri‐infarct for stroke, and impacted core and peri‐impact area for TBI), while the latter presents more widespread cell loss including areas remote from the initial injured brain region. Indeed, the evolution of this remote cell death into secondary cell death after the onset of stroke and TBI has now been recognized to extend outside the brain, specifically to the spleen—a major source of inflammatory response—indicating that peripheral factors contribute significantly to secondary cell death.^4^, ^8^, ^9^ Moreover, the severity of this secondary cell death may be influenced by age, as the young brain, which exhibits more plasticity than the adult brain, may respond more favorably via host brain repair after the insult. Additionally, based on temporal sequence of the cell death cascade of events, the initial insult is usually considered the acute stage, while secondary cell death is viewed as the chronic progression of cell degeneration. Although both stroke and TBI have been traditionally considered as acute brain disorders, accumulating evidence suggests that secondary cell death persists over long‐term, with multiple cell death processes, in particular inflammation, exacerbating these progressive degenerative pathways.^9^, ^10^, ^11^, ^12^ Accordingly, the gradual nature of inflammation presents as an appealing therapeutic target for both stroke and TBI.

## 3 INFLAMMATION PARALLELS SECONDARY CELL DEATH IN STROKE AND TBI

While the central nervous system (CNS) has been previously considered an immune‐privileged system, accumulating evidence advances a dynamic neuroinflammatory interaction involving leukocytes and glial cells.^9^ This aberrant inflammatory response plagues numerous neurological diseases. In stroke and TBI, the initial insult activates an acute inflammatory reaction to combat primary tissue damage, subsequently triggering the secretion of proinflammatory cytokines from resident microglia.^9^, ^13^, ^14^ Additionally, increased permeability of the blood‐brain barrier (BBB) allows peripheral leukocytes to intrude the injured brain.^9^, ^15^, ^16^ Further, sustained microglial activation exacerbates chronic inflammation throughout the CNS, fueling a toxic environment that continuously aggravates secondary axonal degeneration and neuronal death.^9^, ^17^ Consequently, therapeutically suppressing the neuroinflammatory cascade represents a major aim of recent investigative efforts to reduce neurological damage following stroke and TBI.

### 3.1 Acute inflammation in stroke and TBI

Following the onset of stroke, the acute inflammatory phase is characterized by elevated secretion of proinflammatory cytokines interleukin (IL)‐6, IL‐1β, and tumor necrosis factor (TNF)‐⍺ into blood circulation and the cerebrospinal fluid (CSF).^15^, ^16^ While localized upregulation of TNF‐⍺ and IL‐1β has been primarily attributed to microglia 1 (M1) activated microglia, neurons also promote expression of IL‐6.^16^ Moreover, the ischemic microenvironment stimulates microglial elevation of cluster of differentiation 14 (CD14), a pattern recognition receptor on peripheral monocytes and a major component of innate immunity, indicating that resident microglia may mediate the acute inflammatory response following stroke.^17^ These findings provide novel research avenues for recent efforts seeking to convert microglia from the proinflammatory M1 to the neuroprotective M2 phenotype, promoting the secretion of neurotrophic (eg, TGF‐beta) and antiinflammatory (eg, IL‐10) factors aiming to prevent further neuronal loss and facilitate tissue repair.^14^, ^18^ However, there is an increasing trend arguing against classifying inflammatory responses within the constraints of in vitro defined macrophage polarization phenotypes “M1” and “M2.” There is evidence indicating the concurrent expression of both “M1” and “M2” phenotypic markers on the microglia/macrophages, suggesting that the polarization phenotypes cannot be easily confined within this M1/M2 binary nomenclature. Both stroke and TBI may induce a broad spectrum of simultaneous expression responses involving both pro‐ and antiinflammatory reactions, thereby demonstrating a heterogeneous inflammatory response in the injured brain.^19^

The release of cytokines, chemokines, cellular adhesion molecules (CAMs), and matrix metalloproteinases (MMPs) by damaged neurons and auxiliary cells, such as microglia, astrocytes, and neutrophils, amplifies the neuroinflammatory cascade during the subacute phase of ischemic stroke and TBI.^4^, ^16^, ^19^, ^20^ Upregulation of MMPs, in particular, exacerbates localized inflammatory responses by increasing BBB permeability, thereby permitting peripheral leukocytes to infiltrate the injured brain.^19^, ^20^ Additionally, CAMs facilitate leukocyte adherence to cerebral vasculature, allowing further recruitment of cells to the injured area. Activated microglia and astrocytes prolong inflammation into the chronic phase via continued secretion of cytokines, chemokines, and CAMs, thus attracting more peripheral macrophages and neutrophils through the leaky BBB and other novel deleterious microglial and downstream signaling pathways.^21^, ^22^, ^23^ Neuronal loss and cerebral edema may result from this progressive inflammation, compromising brain structure and function.^9^, ^19^, ^20^, ^21^

Similar to stroke, the CNS following TBI undergoes a brief, neuroprotective phase during the acute inflammatory response following initial insult, yet this prosurvival stage is inadequate to provide neuroprotection for lasting inflammation.^12^ The principal injury induced by TBI is physical, involving damage to neurons and disturbances of the BBB.^12^ Following this primary mechanical injury, an acute “neuroprotective” phase and a chronic “neurodegenerative” phase are the two phases of an immune response analogous to those of stroke.^14^ Microglial cells mobilize into a proinflammatory phenotype, and some cells manage regenerative/neuroprotective abilities to combat such injury during the acute stage.^14^ For example, microglia may stimulate principal neurogenesis in the dentate gyrus of the hippocampus and extensive cellular generation.^13^ Yet, the neuroprotection is inadequate because activated microglia that emit proinflammatory cytokines contribute significantly to acute inflammation, as seen with TBI patients possessing activated microglia two decades after initial injury.^12^, ^13^ Additionally, mouse models of TBI display a considerable increase in stimulated microglial cells in both white and gray matter at the TBI affected cortical location and in the neighboring ipsilateral and distal sections.^11^

### 3.2 Chronic inflammation in stroke and TBI

Following acute inflammation, a chronic neurodegenerative phase further contributes to neuroinflammation and coincides with stroke and TBI disease progression, modulated by both central and peripheral immune systems.^9^, ^10^, ^14^ This fragile communication between brain‐resident microglia and systemic lymphocytes must be coordinated correctly,^22^ as disruption of this intricate balance exacerbates central neuroinflammation and, consequently, secondary cell death.

A compromised BBB and tissue damage of the parenchyma and cerebral vasculature is linked with the chronic neurodegenerative phase of stroke.^9^, ^23^ A weakened endothelial cell barrier promotes infiltration of serum proteins and immune cells, contributing to secondary BBB disruption and heightening the original injury induced by stroke.^15^ This worsens physiological damage by elevating cerebral pressure and increasing secondary cell loss.^15^ Ischemic stroke prompts an autoimmune reaction to neuronal antigens that could perhaps escalate or mitigate lasting neuroinflammation.^24^

Akin to the case of stroke, peripheral immune cells infiltrate the TBI brain via the compromised BBB, promoting the secretion of proinflammatory cytokines, immune cell recruitment, and microglial activation. A decline in hippocampal neurons and reduction in cell propagation in the subgranular zone and ipsilateral subventricular zone further extend this vicious cycle of chronic neuroinflammation.^11^ The toxic environment induced by secondary neuroinflammation may contribute to poor graft survival of cell transplants reported in laboratory investigations for cell therapy for TBI.^25^ The secondary cell death cascade induced by chronic inflammation may be the connection between TBI and Alzheimer's disease (AD) neuropathology.^13^ Many characteristic indications of Alzheimer's, specifically amyloid‐beta (Aβ) plaques and neurofibrillary tangles, were discovered in the brains of patients with chronic TBI.^26^ Maturing microglia's reduced phagocytic capability is associated with Aβ42 aggregation and thus, a reduction in microglial elimination of Aβ plaques.^13^ Furthermore, TBI patient brains of all age groups displayed senile Aβ plaques for even children through post mortem analysis, indicating the source of AD to be TBI.^13^ Beyond AD, TBI pathology is also related to many other neurological disorders upwards of 6 months following insult.^27^ Several proteins involved in neurodegenerative disorders have been identified in postmortem TBI brains, produced during 4 hours to 5 weeks postinjury. Aβ plaques increase with beta‐secretase, presenilin 1, and amyloid precursor proteins, and alpha‐synuclein may be found within the axonal bulbs.^28^ Alpha‐synuclein serves as a presynaptic nerve cell protein that accumulates to produce harmful protofibrils,^29^, ^30^ secreted from damaged neurons and also aggregated in the CSF of infant TBI brains.^29^, ^30^ Synucleinopathy also bridges the connection between TBI and Alzheimer's disease to Parkinson's disease (PD). PD demonstrates a similar pathology of microglial activation to dopaminergic neuronal cell loss, characterizing a reactive gliosis which elevates proinflammatory cytokines in the brain and CSF of PD patients.^4^ Lastly, microglia are the earliest to react to a damaging spinal cord injury and may sustain their activation for a minimum of 6 months following injury in humans.^31^ Astrocytes and intraspinal neurons aid in maintaining this immune response by generating proinflammatory cytokine IL‐1β.^31^

### 3.3 Central and peripheral sources of inflammation

Both central and peripheral systems intimately interact in neuroinflammation of CNS disorders, creating a hyperactive immune response that ultimately damages the neural tissue instead of repairing it.^9^, ^32^, ^33^ Following stroke or TBI, central inflammation refers to the involvement of local CNS cells in the inflammatory response, while peripheral inflammation concerns the role of the systemic immune response involving peripheral organs, namely the spleen. Elucidating the central and peripheral sources of inflammation, as well as potential mechanisms of action contributing to this disease progression, is critical in establishing therapeutic strategies to target the secondary cell death cascade in stroke and TBI. Recognizing that the ligand‐receptor pair CCL20‐CCR6 plays a key role in the chemotaxis of dendritic cells, effector/ memory T cells and B cells under homeostatic and inflammatory conditions, including stroke and TBI, we focus this section on CCL20.

CCL20 serves as a chemokine in CCR6 expressing cells. Using an experimental autoimmune encephalomyelitis (EAE) model, an animal model for brain inflammation, CCL20 operates as a ligand for CCR6, allowing homing of lymphocytes along with other leukocytes to neural tissue,^34^ as well as tracking of Th17 or Th1 CD4^+^ cells which produce proinflammatory cytokines contributing to chronic neuroinflammation.^34^, ^35^ The expression of CCL20 in the choroid plexus aids the passage of CCR6^+^ T cells to invade the CNS of the EAE model, further allowing a CCR6 independent pathway of recruitment of T cells to the brain parenchyma.^34^ Further, proinflammatory cytokines IL‐6 and IL‐17 elevate CCL20 expression.^35^

Peripheral involvement in chronic inflammation occurs in stroke. An analysis of the cytokine profile in mice following stroke reveals polarized T‐cell responses dependent on the type of mice used.^36^ C57BL/6 mice display a Th1 polarized response, while BALB/c mice display a Th2 polarized response, indicating that chronic neuroinflammation in stroke patients could arise from peripheral or central involvement depending on the individual.^36^

Similarly, a lateral fluid percussion model of TBI demonstrates that the expression of CCL20 is increased in the spleen and thymus 24 hours postinjury, and in the cortex and hippocampus 48 hours postinjury, indicating a mechanism underlying peripheral involvement in neuroinflammation.^32^, ^33^, ^34^ The expression of CCL20 in the spleen and thymus after TBI before that in the brain, along with reduced brain CCL20 expression following splenectomy, advances a peripheral mechanism of activation for CCL20 upregulation in the CNS.^32^ These findings also implicate the role CCL20 in neuroinflammation following TBI. The increase of CCL20 in the spleen and thymus after TBI may imply that a peripheral signal stimulates neuronal degeneration.^32^ In addition, other studies reveal that the liver may function in worsening the neuronal degeneration after TBI. Deficiency of hepatic Kupffer cells decreases ED‐1‐positive macrophage and neutrophil migration into an IL‐1β‐injected brain.^37^

Peripheral immune and inflammatory systems (ie, spleen) function alongside central inflammation caused by microglia and other inflammatory mediators. Altogether, injury to the CNS induces a peripheral and central immune response contributing to neuroinflammation, resulting in a chronic inflammatory state that exacerbates neural degeneration and retards recovery.

## 4 CELL‐BASED THERAPY FOR STROKE AND TBI

**FIGURE 1:**
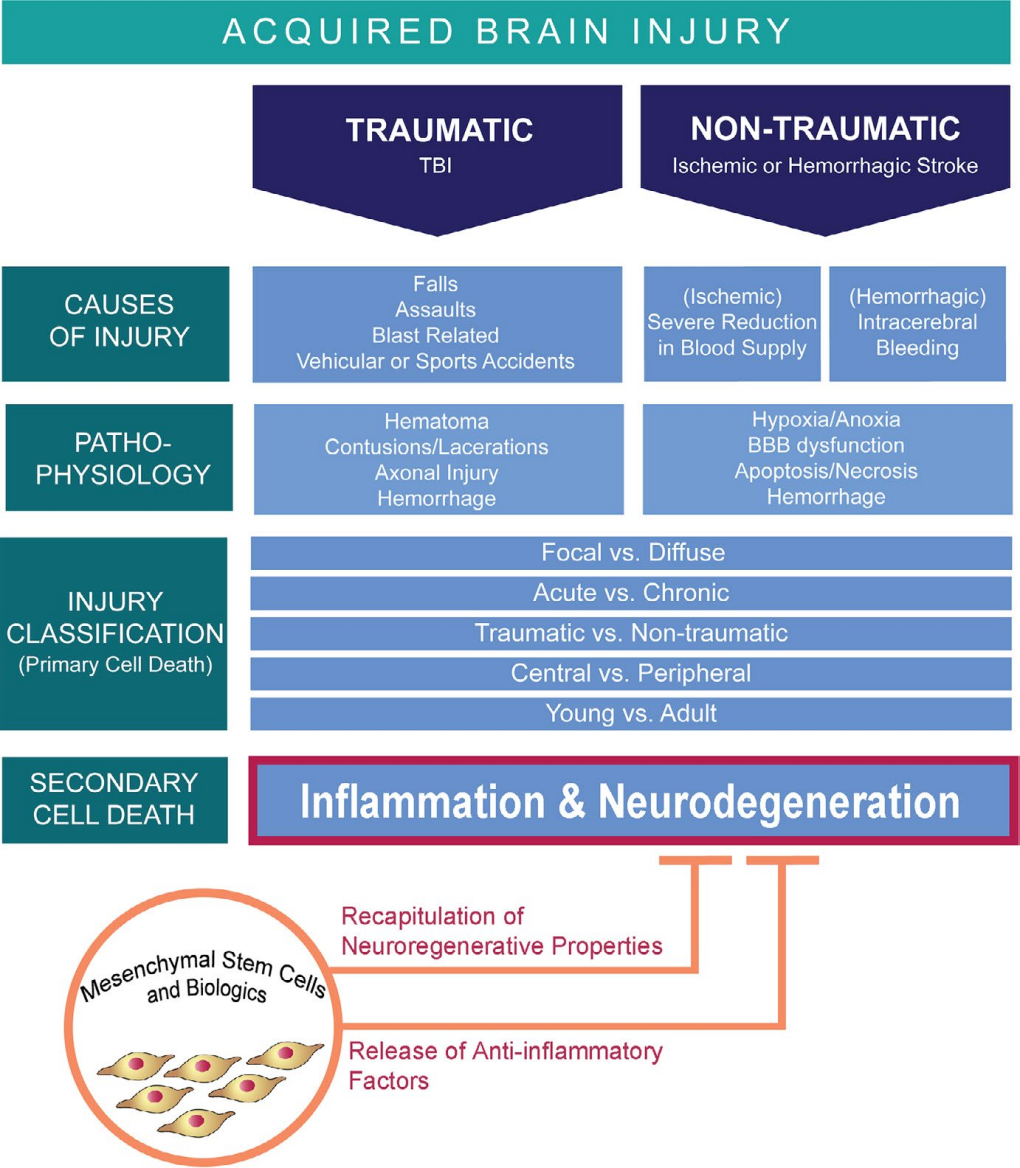
Summary of acquired brain injury causes, pathophysiology, and relation to primary and secondary cell death. Mesenchymal stem cell transplantation stands as an attractive option for attenuating the inflammation induced by stroke and traumatic brain injury (TBI)

The overlapping pathologies of stroke and TBI suggest that therapies that robustly attenuate cell death in stroke may likely prove effective in TBI and vice versa. Of note, cell‐based regenerative medicine is shown to be effective in stroke (Table 1) and TBI, with clinical trials underway for both disease indications.^38^, ^39^, ^40^ As mentioned above, the primary cell death for both ABI disorders may be distinguished as either focal or diffuse, suggesting that the logical target will be localized delivery (intracerebral) and systemic (intra‐arterial or intravenous), respectively. Recognizing that both central and peripheral factors play key roles in the secondary cell death also indicates that direct transplantation and systemic delivery of cells may prove effective. In terms of aging effects on cell‐based therapy, stand‐alone cell transplants may be sufficient to harness the young brain toward regeneration, while providing extra enhancement of the adult brain may be required, such as combining cell transplants with other treatment approaches, such as hypothermia^41^, ^42^ and electrical stimulation,^43^, ^44^, ^45^ in order to facilitate endogenous brain repair mechanisms. Moreover, significant attenuation of both subacute and chronic inflammation may be achievable through cell‐based therapy (Figure 1).^4^, ^19^, ^20^, ^21^ Subacute administration of cells aims to enable neuroprotection and preclude secondary cell death by ameliorating inflammation, apoptosis, mitochondrial dysfunction, and oxidative stress, while chronic delivery is intended to promote neuroregeneration by way of synaptogenesis, neurogenesis, angiogenesis, and vasculogenesis. Stimulation of these regenerative processes can mitigate inflammation and repair the BBB and other cerebral infrastructure.^4^, ^46^, ^47^ The additional knowledge that acute and chronic cell death events may worsen disease outcomes could necessitate initial bolus injection of cells in the early stage, followed by booster transplants at the progressive phases of the disease. A common denominator among these focal and diffuse, central and peripheral, young and adult, and acute and chronic cell death manifestations of stroke and TBI is the occurrence of aberrant inflammation, indicating that cell‐based therapy directed against this secondary cell death mechanism may aid in retarding and even halting the disease progression of stroke and TBI.

**TABLE 1:** Milestone studies of mesenchymal stem cells (MSCs)

Discovery	Future directions	Proposed clinical applications
MSCs derivation from different tissues^98^, ^99^, ^100^, ^101^, ^102^	Vis‐a‐vis comparisons between MSCs derived from different tissues are needed to reveal optimal MSCs	Acute stroke
MSCs display multipotency^103^, ^104^, ^105^, ^106^	Optimization of MSC multipotency	Subacute stroke
MSCs can be primed to differentiate into specific neural lineages^107^, ^108^, ^109^, ^110^, ^111^, ^112^	Optimization of MSC neural differentiation	Chronic stroke
MSCs can be genetically engineered^100^, ^101^, ^113^, ^114^, ^115^, ^116^	Optimization of genetic modification for MSCs	Intravenous delivery
MSCs exert therapeutic effects in cell culture models of stroke^117^, ^118^, ^119^, ^120^, ^121^	Increasing translational potential of in vitro stroke models for testing MSC efficacy	Intra‐arterial delivery
MSCs afford beneficial effects in animal models of stroke^103^, ^104^, ^105^, ^106^, ^122^	Increasing translational potential of in vivo stroke models for testing MSC efficacy and safety	Autologous grafts
MSCs reduce stroke‐induced neuroinflammation^4^, ^123^	Determine specific neuroinflammatory pathway targeted by MSCs	Biomarker, Allogeneic grafts
MSC grafts found to be safe^74^, ^75^, ^77^, ^78^	Long‐term study is needed to determine any tumorigenic risk	Safety measures
MSCs stimulate endogenous neurogenesis^69^, ^124^	Determine specific neurogenic pathway targeted by MSCs	Biomarker
MSCs secrete neurotrophic and neurorestorative factors^125^, ^126^	Determine specific neurotrophic and neurorestorative pathway targeted by MSCs	Biomarker
MSCs can be transplanted intracerebrally or peripherally^116^, ^124^, ^127^	Optimization of route of delivery for MSCs	Multiple cell delivery routes

## 5 TARGETING INFLAMMATION WITH CELL‐BASED THERAPY IN STROKE AND TBI

Many instances of ABI, specifically cases of stroke and TBI, have now been characterized by their inflammatory‐plagued pathology, which further exacerbates secondary cell death progression. This rampant inflammation afflicting stroke and TBI correlates with poor functional recovery and stems from both central and peripheral organs, namely the spleen. Deciphering the origin and mechanisms of this robust inflammatory response provides insight into not only secondary cell death processes that exacerbate tissue damage, but also offers novel therapeutic targets for attenuating stroke and TBI disease progression. Acknowledging the vital role of both central and peripheral systems is paramount in gaining a better understanding of these neuroinflammatory mechanisms, widening our scope for antiinflammatory strategies for stroke and TBI. Moreover, the spleen serves as a key peripheral organ contributing to the systemic inflammatory response, identifying it as a prime target for examining these secondary cell death cascades.

### 5.1 Bone marrow–derived mesenchymal stem cells for ABI

Key lab‐to‐clinic translational factors, including dosage, timing, and route of administration, influence the success of a cell transplant, particularly regarding its capacity to counter neuroinflammation following stroke and TBI.^48^ However, the specific cell type used may have the most determinative power for cell transplantation efficacy. Various cell types ranging from embryonic to engineered cells have been explored as cell donors for transplantation in stroke and TBI. Although the pluripotency and multipotency endowed to embryonic/fetal stem cells has previously established them as the yardstick of “stemness,” these cells present notable ethical and safety considerations regarding the source from which they are harvested and high risks of tumorigenicity. Due to these logistical concerns, focus has shifted to other types of cells, specifically adult tissue‐derived cells. While most scientists concur that bone marrow–derived mesenchymal stem cells (BM‐MSCs) do not fit the exact definition of “stem cells,” they do display a similar capacity for brain repair.^48^ BM‐MSCs’ adult tissue origin, robust safety profile, availability, neuroprotective and regenerative effects, and established research history advances them as an attractive candidate for cell‐based therapy.

In in vitro and in vivo studies of stroke, BM‐MSCs afford robust functional recovery in many models of brain disorders, promoting them as an attractive translational cell product.^48^ In animal models, BM‐MSC transplantation decreases brain damage and ameliorates motor and cognitive performance. In a hemorrhagic stroke rat model, transplanted BM‐MSCs reduce inflammation after intraventricular infusion of BM‐MSCs, resulting in decreased proinflammatory cytokine expression levels, including IL‐6, IL‐1α, and IFN‐γ.^49^ Furthermore, present evidence suggests that the release of growth factors by BM‐MSCs or their neurotrophic exosomes, direct cell replacement, and encouragement of endogenous brain repair processes such as angiogenesis, neurogenesis, and synaptogenesis may be responsible, although the precise mechanism has not yet been identified.^48^ Unfortunately, specific study design limitations such as underpowered trials, challenges in patient recruitment within the targeted disease phase, and inferior brain imaging capabilities, among others hinder mechanism‐based analyses. Initially, studies visualized cell effects with a reductionist ligand‐receptor model to determine the restorative mechanism of cell implantation, but this was later shown to be overly simplistic.^48^ With scarce evidence supporting mesenchymal stem cell MSC differentiation, the alternative bystander effect mechanism of grafted cells has been proposed, including secretion of growth factors that confer antiinflammation, antioxidative stress, antiapoptosis, and neurogenesis, altogether acting synergistically to deliver a therapeutic outcome. The intricate pathology of stroke illustrates the need to optimize the cell transplant regimen either as a stand‐alone or an adjunctive treatment with other standard stroke therapeutics in order to facilitate maximum functional benefits. Therefore, combined treatments, including biomaterials, pharmaceutical utilization, and transplantation of additional cell types, may be principal in guaranteeing the use of the optimal therapeutic profile for patients being treated with cell therapy.^48^ Combined BM‐MSCs and peroxisome proliferator‐activated receptor gamma (PPARγ) agonist pioglitazone (PGZ) produce antiinflammatory effects.^50^ PGZ‐treated rats exhibit enhanced PPARγ expression following their attraction of allogeneic BM‐MSCs, demonstrating the relationship between PPARγ and BM‐MSCs.^50^ Furthermore, male stroke rats display significantly reduced expression of inflammatory IL‐6 and caspase‐3, suggesting that combined therapy for BM‐MSCs with PGZ counters neuroinflammation.^50^ Additionally, administering regulatory T cells (T_regs_) with BM‐MSCs attenuates inflammation more effectively than BM‐MSCs alone, emphasizing BM‐MSC combined therapy's potential for stroke treatment.^51^ Altogether, BM‐MSCs in combination with adjunctive therapies may provide enhanced functional recovery in inflammation‐mediated neurodegenerative stroke models.^12^, ^52^, ^53^, ^54^, ^55^, ^56^, ^57^, ^58^, ^59^

As previously discussed, sustained and pervasive inflammation not only largely underlies secondary cell death after ischemic stroke, but also following TBI. Thus, knowledge of the antiinflammatory and immunosuppressive properties of BM‐MSCs has spurred recent preclinical investigation of BM‐MSC transplantation as a treatment for TBI. Indeed, intravenous infusion of BM‐MSCs in TBI rats has been observed to reduce the number of local microglia and peripheral immune cells at the infarct region.^60^ Potentially via its suppression of microglial activation, BM‐MSC treatment correlates with significant downregulation of proinflammatory cytokines IL‐1β, IL‐6, IL‐17, TNF‐α, and interferon gamma, as well as with elevated expression of antiinflammatory cytokines IL‐10 and transforming growth factor‐β1.^61^ In line with these findings, further data suggest that transplanted BM‐MSCs dampen phagocytic activity and stimulate polarization of microglia to the more neuroprotective and antiinflammatory M2 phenotype, thereby improving functional deficits in TBI rats.^62^ Moreover, BM‐MSCs may also enhance functional recovery by differentiating into neurons and astrocytes,^63^ as well as via trophic support of endogenous neural regeneration.^64^, ^65^

While yielding favorable results without modification of the naive cell phenotype, augmenting the properties of BM‐MSCs may increase their efficacy in treating TBI. For example, the homing accuracy of transplanted BM‐MSCs to TBI‐induced lesions has been markedly improved by stimulating these cells to excessively produce fibroblast growth factor 21.^66^ Furthermore, in other current TBI rat models, genetically modifying BM‐MSCs to overexpress the antiinflammatory cytokine IL‐10 has not only been associated with increased autophagy and mitophagy—which indicate the protection of neural cells from inflammation^67^—but also with enhanced immunomodulation concurrent with superior functional recovery compared to unmodified BM‐MSC treatment.^68^ Thus, although BM‐MSC transplantation alone may be sufficient to provide neuroprotection, advancing these cells as a treatment for TBI may be improved by modifying these cells to optimize their therapeutic properties in the injured brain.

Accumulating evidence shows that the therapeutic effects of MSCs in different pathologies may be mediated by the paracrine secretion of a broad array of biological active molecules.^61^, ^69^ The secretomes and exosomes containing those molecules can be easily isolated from the cells and used as a biodrug.^70^, ^71^ In the case of neuroinflammation‐related diseases, the intranasal administration of the secretome has been evaluated as a noninvasive and effective route to reach the brain.^72^, ^73^ Due to the translational value of this cell‐free strategy, the use of secretomes and exosomes derived from MSCs warrants further investigation in the treatment of ischemic stroke and TBI.

### 5.2 Clinical perspective on MSC therapy for stroke and TBI

Accumulating preclinical evidence supports the therapeutic potential of BM‐MSCs as a feasible model for cell‐based therapy for CNS disorders, yet their efficacy and viability in the clinic still face significant questions and challenges. Moreover, a limiting factor in the clinic may be the collection of bone marrow which presents as a painful procedure that involves large needle aspirates. In preclinical studies, transplanted BM‐MSCs have consistently delivered auspicious outcomes in animal models, yet their functional effects have differed notably between studies, ranging from the secretion of trophic factors to the mobilization of endogenous stem cells.^48^ In light of such discrepancies, analyzing the effects of BM‐MSC transplantation in human subjects of recent clinical trials may more effectively indicate the utility of this cell‐based therapy as a future treatment for ischemic stroke and TBI.

Clinical trials in stroke reveal no ill effects and ameliorated neurological outcomes as measured by the Rankin scale and Barthel index after delayed autologous transplantation (primary infusion at 4 weeks after stroke) of 100 million MSCs (SH‐2 and SH‐4 positive) in five stroke patients.^74^ However, these functional benefits significantly declined 12 months after transplantation.^74^ Moreover, a similar autologous intravenous bone marrow transplantation administered 7‐10 million per kilogram of bone marrow–derived mononuclear cells (BM‐MNCs) 24 and 72 hours after stroke.^75^ While BM‐MNCs are distinct from BM‐MSCs, they often contain a small percentage of BM‐MSCs.^76^ BM‐MNCs have been used more often than BM‐MSCs in clinical trials due to their comparative availability.^76^ This trial resulted in significant improvements on the modified Rankin scale, Barthel index, and National Institutes of Health Stroke Scale (NIHSS) without any ill effects over a 6‐month trial for the majority of patients who received the transplant.^75^ Following these positive results, a blinded outcome assessment in India with 120 patients used phase II, multicenter, and parallel groups in a randomized trial of BM‐MNCs.^77^ Fifty‐eight stroke patients who received a mean of 280.75 million BM‐MNCs at a median of 18.5 days poststroke exhibited no changes in modified Rankin scale shift analysis, Barthel index score, NIHSS score, and infarct volume as compared to nontransplanted stroke patients at 6‐months after transplantation.^77^ These results suggest that although intravenous transplantation of BM‐MNCs may be safe, BM‐MNCs may lack effectiveness for subacute stroke. Another trial investigated a smaller subpopulation of CD34^+^ BM‐MNCs in stroke patients for their therapeutic potential.^78^ Using intra‐arterial administration of 100 million autologous, immunoselected CD34+ stem/progenitor cells in five stroke patients within 7 days after severe anterior ischemic stroke (NIHSS score ≥ 8) manifested improvements in NIHSS score and in the modified Rankin scale.^78^ Additionally, there was a reduction in lesion volume over a 6‐month follow‐up period.^78^ As there were no adverse effects, this investigation furthered evidence indicating the safety of using intra‐arterial delivery of BM‐MNC CD34+ cells for stroke therapy.^78^ Taken together, these studies seem to indicate that BM‐MNCs are safe and may have promising short term and long‐term effects.

Upon critical examination, clinical trial results demonstrate that BM‐MSC and BM‐MNC transplantations may act as efficient biocompatible procedures for treating stroke. However, more extensive examination of the procedures is necessary because these studies largely employ small sample sizes and an open‐label designation. Optimization of cell dose, route, and timing of delivery, with special attention to comparing these between different cell types and strains, is warranted to improve therapeutic outcomes of cell therapy. Moreover, adherence to STEP guidelines will undoubtedly increase the rigor in study design, data analysis, and reporting. With the recent positive clinical trials of endovascular thrombectomy in acute ischemic stroke, the potential of cell therapy as an adjunctive treatment alongside thrombectomy or thrombolysis may further enhance its successful translation to the clinic. Furthermore, disparities among the trials may be explained by extensive investigation of the methods, which may make cross‐study comparisons more difficult. Indeed, the treatment plans and specific cell types selected for transplantation differ widely between the preclinical and clinical transplant groups. As previously noted, the identity of the donor cell may have substantial influence on the result of cell therapy, and each of these clinical trials utilized different donor cells. For instance, Savitz and colleagues utilized a widespread group of antibodies for flow cytometry—CD3, CD14, CD16, CD19, CD20, CD34, CD45, CD56, Lin 1, CD133‐2—while Bang and colleagues employed Src homology 2‐ and Src homology 4‐type cells.^74^, ^75^ Echoing this, Prasad and collaborators also distinguished the BM‐MNCs through flow cytometry and only employed CD34 and CD45.^77^ However, a magnetic cell isolation process was utilized in the study conducted by Banerjee and team in order to define purified CD34+ cells alone.^78^ Since the type of donor cell differs in each trial, cross‐comparisons become more difficult to conduct. Moreover, the timing of intervention varies in each of the four trials: 4 weeks in Bang's trial, 1‐3 days in Savitz's examination, 18.5 days in Prasad's investigation, and within 7 days since the onset of stroke in Banerjee's experiment.^74^, ^75^, ^77^, ^78^ Likewise, the method of treatment delivery varied in each trial, as Bang, Savitz, and Prasad employed an intravenous route while Banerjee utilized an intra‐arterial route.^74^, ^75^, ^77^, ^78^ Along with the inconsistencies enumerated above, each trial failed to use an appropriate dosage. As indicated in multiple preclinical studies of cell transplants, a dose range of 4 million cells in a 250 g rat or 840 million cells in a 75 kg human administered intravenously is most effective.^79^ In these clinical trials, Bang and colleagues employed 100 million, Savitz employed a mean of 600 million, Banerjee used 100 million, and Prasad utilized 280.75 million.^74^, ^75^, ^77^, ^78^ Thus, the doses used in these trials were substantially lower compared to the threshold of an efficient dose, with exception to Savitz's experiment in which a dose closer to the threshold was used.^75^ Nonetheless, Savitz and team conducted an open‐label trial, so even though the patients participating in the trial demonstrated recovery, their results may have limited validity.^75^ An evaluation of the literature, examining the specific donor type in each trial illuminates the scant number of studies that describe these cells’ safety, efficiency, and method of action with respect to the Stem cell Therapeutics as an Emerging Paradigm for Stroke (STEPS) lab‐to‐clinic translational guidelines.^79^ Furthermore, if the STEPS guidelines are followed and clinical trial procedures are designed based on laboratory science, future studies investigating the clinical administration of MSCs will likely improve therapeutic outcomes.^79^, ^80^, ^81^

In the context of clinical studies of MSC therapy for TBI, a 2013 trial enlisting 97 TBI patients administered autologous BM‐MSCs via lumbar puncture supports the safety and efficacy of this cell therapy.^82^ Approximately 40% of patients demonstrated improved neurological function following transplantation.^82^ Of 73 patients presenting with motor disorders, twenty‐seven displayed enhanced motor improvements.^82^ The study noted variable outcomes depending on factors such as the administrative window postinjury and patient age, with younger patients more responsive to the cell transplant benefits.^82^ In addition, BM‐MNCs have also advanced to clinical trials for TBI. Twenty‐five patients receiving intravenous delivery of BM‐MNCs in a dose escalation design (6, 9, 12 × 10^6^ cells/kg body weight) presented no severe adverse effects.^83^ Moreover, BM‐MNC treatment correlated with a downregulation of inflammatory cytokines IL‐1β and IFN‐γ, paralleling preclinical evidence in animal models of TBI.^83^ These findings support the safety and logistical feasibility of BM‐MNC transplantation for TBI.

Current efforts reveal novel information about the interactions between endogenous or grafted cells and immune cells.^84^, ^85^, ^86^, ^87^ Surrounding populations of adaptive (B and T cells) and innate immune cells (monocytes, macrophages derived from monocytes, microglia) regulate cell and noncell autonomous mechanisms, which has broad implications for regenerative medicine.^84^ Indeed, the infiltrating circulating population of immune cells initiates many coincident postinjury immune responses, some of which increase or reduce inflammation. This, in turn, triggers an immune response from grafted cells.^85^, ^86^ For example, coculturing macrophages expressing MHC II with adipose tissue‐derived MSCs demonstrated that MHC II upregulates collagen settlement and accelerates expression and proliferation of MMPI, PLOD2, and PTGS2.^86^ MMPI incites migration of cells, PLOD2 plays a vital role in maintaining intermolecular cross‐links, and PTGS2 regulates the proinflammatory immune response.^86^ The adaptive immune system, however, hinders the grafted cells’ therapeutic and antiinflammatory potential in a different manner.^87^ Even though the host immune system tolerates grafted cells, T cells and NK cells may not, and may attack cells such as NSCs due to their MHC I expression, which, in turn, may induce immune‐mediated cytolysis.^87^ In this regard, however, MSCs may be able to regulate both naïve and memory T‐cell response, despite a deficiency in CD4^+^/CD25^+^ T_regs_ or antigen‐presenting cells (APCs) in the MSC culture.^87^ Although the cellular processes behind the interactions among immune and grafted cells require further investigation, evidence suggests that this interlinkage may be double‐edged. The influence of this interlinkage has positive and negative implications on grafted cells' viability and excretion of trophic elements and their use for stroke and TBI.

## 6 CONCLUSION

Stroke and TBI, two principal forms of ABI, pose a significant health and economic burden globally, and limited treatment options necessitate a novel therapeutic strategy to attenuate disease progression.^88^, ^89^, ^90^, ^91^ Primary cell death directly results from stroke or TBI, and the extent of this, brain damage is categorized as either focal or diffuse. Along with the influence of patient age, central and peripheral sources of immune cells prominently contribute to secondary neurodegeneration during both the acute and chronic phases following stroke and TBI.^92^, ^93^, ^94^, ^95^, ^96^, ^97^ Indeed, neuroinflammation stands as the common denominator that accompanies both disease pathologies and closely parallels secondary neural cell loss throughout their progression. As such, elucidating the dynamic involvement of both central and peripheral sources, especially the interplay between the brain and the spleen, is key to understanding the mechanisms underlying neuroinflammation. MSC transplantation as a regenerative biologic therapy targeting this deleterious inflammation has emerged as an innovative approach. Clearly, optimization of the cell therapy approach is warranted that will allow the transplantation strategies to accommodate the variable brain inflammatory responses. Localized intracerebral delivery of MSCs may be more appropriate for focal injury as this will directly target the inflamed area. In contrast, systemic delivery may be utilized for diffuse brain damage, as well as targeting the peripheral component such as the spleen, in order to fully retard both central and peripheral sources of inflammation. Additionally, while stand‐alone MSC administration may be sufficient to confer neuroprotection and rejuvenation in younger brains, combination treatments may be necessary to parallel the gold standards of treatment, including rehabilitation therapy. Moreover, the initial MSC injection during the acute phase may need to be supplemented later with repeated MSC infusions throughout the chronic phase following stroke or TBI. Due to their immunomodulatory and antiinflammatory properties, as well as their long‐standing safety profile, BM‐MSCs stand as a favorable cell therapy model for transplantation. Accumulating preclinical evidence thus far supports their therapeutic potential for stroke and TBI. Strict adherence to STEPS guidelines, such as incorporation of randomization, blinding and sample size calculations into study design, use of comorbid animal strains, both male and female animals, investigation of appropriate dose‐response relationships and testing MSCs in at least two animal models, and in independent laboratories will likely generate a rigorous the preclinical framework for the design of safe and effective clinical application of MSCs. Targeting neuroinflammation via MSC therapy represents a novel avenue for future therapeutic endeavors aimed to treat stroke and TBI.

Mesenchymal stem cell transplantation stands as a promising therapeutic approach for many types of acquired brain injury ABI, including ischemic stroke and traumatic brain injury TBI. These injuries presently possess very limited treatment options, especially those that may address the overarching problems caused by primary and secondary cell death. Stem cells, especially bone marrow–derived mesenchymal stem cells BM‐MSCs, are poised to fill this treatment gap, on the basis of their robust neuroprotective effects in the short term, and neuroregenerative and immunomodulatory effects in the long term. Successful translation of MSC transplants from the bench to the bedside may greatly attenuate the immense health and economic burdens posed by stroke and TBI. BM‐MSCs present a particularly attractive option due to their positive preclinical results, relative availability, and well‐established safety record in their use for other diseases. There exist a relatively small number of clinical trials for BM‐MSCs for stroke and TBI, but most have yielded quite auspicious results. However, some questions remain due to one null stroke trial and the limited capacity for cross‐comparison between trials due to disparate designs and outcome measures. This review emphasizes the need for more clinical trials, especially those with a randomized and blinded design and with appropriately scaled MSC dosages. At present, scaling up MSCs remains a rate‐limiting step in some applications, and thus, these findings advance the importance of addressing this issue. Furthermore, in the case of stroke, clinical translation may also be aided by stricter adherence to the recommendations set forth by the STEPS translational guidelines. In addition, this review also brings attention to the emerging interest in MSC transplants administration, including the timing, dosage, number of doses, patient characteristics, and injury specifics, as well as possible administration with attention to or in concert with the innate and adaptive immune system of the patient. MSC transplants as therapeutics for stroke and TBI have developed rapidly over the past decades and will likely only continue to grow. Significant milestones may be reached upon the discovery of viable strategies to improve cell proliferation as well as the progression of MSC transplants to become an accepted standard of care for ABI disorders. MSC therapy as a stand‐alone or combination treatment creates a nearly limitless set of research opportunities. As such, MSC therapy represents a fertile area for future research, and it is likely that many cell‐based innovations and translational applications will continue to manifest in the coming years. The envisioned product is a safe and effective cell therapy designed to abrogate the inflammation‐plagued secondary cell death associated with ABI.

## CONFLICT OF INTEREST

CVB declares patents and patent applications related to stem cell therapy. Additionally, CVB was funded and received royalties and stock options from Astellas, Asterias, Sanbio, Athersys, KMPHC, and International Stem Cell Corporation; and also received consultant compensation for Chiesi Farmaceutici. The other authors have no other relevant affiliations or financial involvement with any organization or entity with a financial interest in or financial conflict with the subject matter or materials discussed in the manuscript apart from those disclosed.
